# Strengthening Chronic Disease Prevention Programming: the Toward Evidence-Informed Practice (TEIP) Program Assessment Tool

**DOI:** 10.5888/pcd10.120106

**Published:** 2013-05-30

**Authors:** Dayna Albert, Rebecca Fortin, Anne Lessio, Christine Herrera, Barbara Riley, Rhona Hanning, Brian Rush

**Affiliations:** Author Affiliations: Dayna Albert, Rebecca Fortin, Anne Lessio, formerly Ontario Public Health Association, Toronto, Ontario; Christine Herrera, University of Toronto and formerly Ontario Public Health Association, Toronto, Ontario; Rhona Hanning, University of Waterloo, Waterloo, Ontario; Brian Rush, University of Toronto, Toronto, Ontario.

## Abstract

Best practices identified solely on the strength of research evidence may not be entirely relevant or practical for use in community-based public health and the practice of chronic disease prevention. Aiming to bridge the gap between best practices literature and local knowledge and expertise, the Ontario Public Health Association, through the Toward Evidence-Informed Practice initiative, developed a set of resources to strengthen evidence-informed decision making in chronic disease prevention programs. A Program Assessment Tool, described in this article, emphasizes better processes by incorporating review criteria into the program planning and implementation process. In a companion paper, “Strengthening Chronic Disease Prevention Programming: The Toward Evidence-Informed Practice (TEIP) Program Evidence Tool,” we describe another tool, which emphasizes better evidence by providing guidelines and worksheets to identify, synthesize, and incorporate evidence from a range of sources (eg, peer-reviewed literature, gray literature, local expertise) to strengthen local programs.

The Program Assessment Tool uses 19 criteria derived from literature on best and promising practices to assess and strengthen program planning and implementation. We describe the benefits, strengths, and challenges in implementing the tool in 22 community-based chronic disease prevention projects in Ontario, Canada. The Program Assessment Tool helps put best processes into operation to complement adoption and adaptation of evidence-informed practices for chronic disease prevention.

## Background

Closing the gap between research knowledge and daily operation of community-based programs and policies is a central concern in public health and chronic disease prevention. Key reviews (1–3) and seminal reports in the peer-reviewed literature (4) call for incorporation of more research evidence into public health practices and policies. Although few argue with the importance of more closely aligning practice and policy with the best available research evidence, the processes required to accomplish this alignment continue to be explored. Although conceptual frameworks have been developed to advance evidence-informed practice (4–7), their application in multiple populations and settings must be enhanced (4).

Best practices may be derived by synthesizing the results from a pool of rigorous research studies that demonstrate the effectiveness of a program for a given health problem or public health concern. The use of the term “best” is derived from evidence-based medicine and the levels of evidence paradigm articulated by Sackett (8). Although synthesis methods have expanded substantially in the last 5 to 10 years, the traditional systematic review tended to emphasize studies using randomized controlled trials (RCTs) (9). Although the RCT is often referred to as the “gold standard” for scientific evidence of causality, the scope and complexity of public health interventions do not always lend themselves to an RCT design (9). Furthermore, experimental studies and systematic reviews can be limited in terms of generalizability and applicability of the findings (9,10).

The concept of “promising practices” in public health emerged to recognize interventions achieving local success in the absence of sufficient research and evaluation resources to be labeled a best practice (6,11–13). Many criteria and definitions of promising practices in public health practice have emerged. In addition to criteria for program effectiveness, plausibility and practicality have been added to the formal identification of best practices (12), as have considerations for underlying health promotion values and goals, theories and beliefs, and understanding of the broader external environment (6). Plausibility considers theoretical underpinnings (such as behavior change principles), evaluability, program logic, and local context as it relates to the needs of intended program users. Practicality considers program fit with the local political environment and available resources (12). Emphasis on plausibility and practicality criteria acknowledge the inherent value of programs not reported in peer-reviewed literature and the need to consider broader contextual issues, including local resource limitations, in developing and designing effective chronic disease prevention programs (14–17).

Adaptation frameworks (17,18) have been developed to provide guidance in adapting a best or promising practice to local conditions without compromising effectiveness, recognizing the importance of supporting the use of best practices in a new community or context (19,20). The concept of “best processes” has been offered to help expand the definition and use of best practices (16). The idea of best processes moves beyond research processes to draw attention to foundational tenets in health promotion practice and calls for continual program reflection and adaptation (6,16,21).

In 2005, the Ontario Public Health Association (OPHA) received provincial funding to develop better processes to support evidence-informed practice at the local level, extending previous work focused solely on dissemination strategies (22). This initiative became known as TEIP (Toward Evidence-Informed Practice). TEIP is a set of tools that aims to support effective health promotion and disease prevention programming by strengthening local capacity for evidence-informed practice. The TEIP Program Assessment Tool aims to strengthen existing programs and to improve the planning of new programs. This article describes the Program Assessment Tool, its application in 1 community, and its relevance to community-based health promotion and chronic disease prevention practitioners.

## The Program Assessment Tool

The primary purpose of a program assessment is to strengthen existing programs through use of a structured review and rating process. The process was built on review criteria used previously to identify best and promising practices in health promotion and chronic disease prevention (12). Developed through an iterative process from 2005 through 2009, the rating process makes explicit the program’s underlying theories or assumptions, identifies areas of improvement in the program’s processes and procedures, and determines program evaluation needs. The tool is not intended to label a program as best or promising but rather to highlight program strengths and areas for enhancement and to provide realistic recommendations for improvement. In this way, the Program Assessment Tool supports continuous program improvement while respecting local experience, wisdom, and context.

The 19 review criteria (Table 1) comprise 4 categories: program need (2 criteria), program content (5 criteria), program process (6 criteria), and program evaluation (6 criteria). The criteria are made more explicit with a set of indicators and a 3-point achievement scale.

**Table 1:** Program Assessment Tool Review Criteria, the Toward Evidence-Informed Practice Initiative, Ontario, Canada

Review Criterion	Description
**Program Need**	
1. Needs assessment	The program responds to demonstrated wants and/or needs of the primary audience.
2. Duplication avoidance/environmental scan	The program fills a unique need in the community/setting that is not met by other programs/services.
**Program Content**	
3. Theory and literature evidence	The program is informed by appropriate theoretical concepts (eg, behavior change theory, social learning, risk reduction) and credible, relevant, and realistic sources of evidence (eg, critically appraised academic literature, credible gray literature, expert advice).
4. Program objectives and logic model	The program has appropriate SMART objectives (Specific, Measurable, Appropriate, Realistic, and Timed) as part of its program logic model.
5. Environmental support	The program creates physical and social environments that support healthy behaviors (eg, walking trails, bicycle racks at worksites, healthy food choices in restaurants and vending machines).
6. Policy	The program develops and, as appropriate, implements policy. Policy refers to changing formal or informal rules of governing bodies to support healthy behaviors, both development and implementation. Policy efforts can be directed at the municipal level (eg, by-laws) and/or the institutional level (eg, school or worksite policy).
7. Sequencing	The program is sequenced appropriately. Sequencing refers to the building of program activities on each other over time, to maximize population impact (eg, awareness, skill building, environmental support, policy development).
**Program Process**	
8. Collaboration	The program can be described as collaborative. There is active involvement of local individuals, groups, and intended audiences in program planning and implementation. The right partners are engaged.
9. Mobilization of community resources	The program identifies and uses resources from within the community/setting.
10. Community engagement	The program engages individuals from the community or setting with the objective of consulting, animating, or sensitizing them to the issue (ie, fostering community buy-in).
11. Sustainability	The program, or aspects of the program, can be maintained in the community or setting over time, without dependence on “one-time” or special resources. The Heart Health Resource Centre’s Sustainability Model considers 4 components: issue, program, behavior change, and partnerships.
12. Visibility	The program demonstrates widespread promotion of the program in the community or setting, and/or those delivering the program are highly visible.
13. Opinion leader support	The program demonstrates or has the potential to elicit the active support and buy-in of formal or informal opinion leaders in the community or setting where it is delivered.
**Program Evaluation**	
14. Formative evaluation/pilot testing/focus testing	The program uses formative evaluation (eg, focus groups, structured surveys, key informant interviews, pretesting) to assess the relevance, comprehension, and acceptability of activities, materials, and methods for the intended audience or population of interest.
15. Process evaluation	The program uses process evaluation to gather feedback, demonstrating that intended audiences were reached and the program was implemented as planned.
16. Outcome evaluation	The program evaluates outcome. It assesses the extent to which the program met stated goals and objectives, as assessed by impact and/or outcome evaluations (eg, changes in behaviors, policies, or supportive environments).
17. Program documentation	The program can be replicated at the same and/or new locations. The presence of program implementation guidelines is a necessary condition for meeting this criterion.
18. Context documentation	The program documents its context completely. The extent to which community-specific contextual factors have been analyzed and documented (eg, relevant municipal policies, local demographics, regional socioeconomic factors, profile of intended audience).
19. Cost-benefit	The program weighs the costs against the benefits and concludes that the potential impact (benefits) of the program is worth the estimated costs.

Program assessment consists of 5 steps: 1) selecting a program for assessment and assigning roles, 2) gathering information, 3) assessing the program, 4) achieving consensus, and 5) planning for program enhancement. A description of the guidelines and accompanying worksheets used in each step are outlined in Table 2. In addition, an online learning tutorial describing use of the tool is available. The Tool’s worksheets and related resources are available upon request.

**Table 2:** Program Assessment Tools Guidelines, Worksheets, and Intended Results, the Toward Evidence-Informed Practice Initiative, Ontario, Canada

Guidelines	Worksheets	Intended Results
1. Select program and assign roles	Roles assignment worksheet	• Program needing improvement is identified • Appropriate program reviewers are identified
2. Gather program information: program informant documents current program activities in 4 categories: program need, program content, program process and program evaluation	Program information survey	• Complete set of program documentation is available • Information on how program addresses 19 evidence-based criteria ready to forward to reviewers
3. Assess the program: independent reviewers assess the program using information collected in the survey against corresponding “best and promising practices” criteria	Program assessment worksheet	• Initial assessment of program against 19 evidence-informed criteria is completed • Draft list of program improvements is identified
4. Achieve consensus: conduct a consensus meeting, whereby reviewers discuss and reach consensus on a rating and suggestions for program enhancement for each criterion	Consensus summary sheet	• Areas for program improvement are agreed upon by all reviewers and understood by program staff
5. Select suggestions to implement: program stakeholders select priority program enhancements and develop a work plan for implementation	Program enhancement work plan	• Feasible plan to implement priority program improvements is developed

### Step 1: Select program and assign roles

Program assessment is a generic process that can be applied to a range of community-based health promotion initiatives. Programs that most benefit from the full assessment process tend to be established programs that are not achieving expected outcomes or have not been updated.

**Figure 1:**
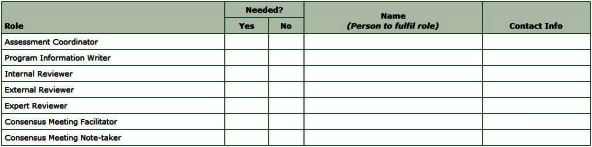
The worksheet summarizes, for each role needed within an assessment, the person responsible and the person’s contact details.

A program assessment requires at least 4 participants: 1 to gather information about the program and 3 to independently review the program information against the review criteria. Three types of reviewers are recommended: a local program stakeholder known as the internal reviewer, an external reviewer, and an expert reviewer who has knowledge of the audience or the initiative under review. If resources permit, 3 additional roles are recommended: an assessment coordinator, a consensus meeting facilitator, and a consensus meeting note taker (Figure 1).

### Step 2: Gather program information

Once a program has been selected and roles assigned, a program informant, usually the manager or coordinator of the program under review, completes the program information survey, which consists of 19 questions designed to document how the program addresses each of the 19 review criteria. Gathering program information can take several hours to several days, depending on how well program records have been maintained. Many past participants have reported that simply completing the program information survey leads to useful insights for improving program processes. Once completed, the survey becomes a valuable source of program documentation, especially in situations where record keeping was previously inconsistent (Figure 2).

1. **Theory and Evidence:**
  Describe any health promotion theories used to inform program design (eg, relevant theories, conceptual frameworks) as well as relevant and credible sources of evidence (eg, critically appraised academic literature, credible grey literature and expert advice). How recently was this information gathered? Please describe relevant examples of how the research, including theory or a conceptual framework, informed program development and implementation.
2. **Program Objectives and Logic Model:**
  List all program level and activity level objectives, whether process or outcome-related. Identify those which are SMART (eg, Specific, Measurable, Achievable, Realistic and Timely). If a program logic model exists, please provide.
3. **Environmental Support:**
  To what degree, if any, has your program addressed Environmental Support to create physical and/or social environments that support healthy behaviours (eg, walking trails, bicycle racks at worksites, etc)?
4. **Policy:**
  To what degree, if any, has your program addressed policy (i.e. changing the formal or informal rules of governing bodies to support healthy behaviours)? Some examples are advocacy to support by-law change or worksite policy development.
5. **Sequencing:**
  Does this program involve a sequence of activities designed to maximize population impact over time (i.e. activities in program are sequenced to move from Awareness to Skill Building to Environmental Support to Policy Development)? Please explain.

**Figure 2.** The program information survey directs the process of collecting program information. This excerpt details 5 (theory and evidence, program objectives and logic model, environmental support, policy, and sequencing) of 19 review criteria.

### Step 3: Assess the program

In the third step, the program information survey and supporting documentation are assessed by 3 reviewers, each of whom may have a different perspective. The internal reviewer is knowledgeable of the program under review. External reviewers, of which there are usually 2, can be a public health colleague, a program stakeholder, or someone with expert knowledge of the type of program or the intended audience.

Reviewers use the program assessment worksheet to document levels of achievement and suggestions for improvement for each of the 19 criteria. There are 3 levels of achievement. Level A indicates substantial room for improvement, level B indicates some room for improvement, and level C indicates the criterion has been satisfactorily addressed and there is little room for improvement. The counterintuitive rating scheme (ie, level C indicates more achievement than level A) endeavors to decrease the unintentional perception of being judged and to increase the positive intention of developing suggestions to strengthen the program (Figure 3).

6. **Environmental Support:**
  The program creates physical and social environments that support healthy behaviours (eg, walking trails, bicycle racks at worksites, healthy food choices in restaurants and vending machines etc).
  - Identified opportunities for creating physical and/or social environments within program but no plan to address them.
  - A plan to address physical and/or social environments is available and appropriate partners engaged by no action yet taken on the plan.
  - Appropriate partners engaged and action taken to address physical and social environments.
**Level:**
**Rationale:**
**Suggestions for Enhancement:**
7. **Policy:**
  The program develops and, as appropriate, implements policy. Policy refers to changing formal or informal rules of governing bodies to support healthy behaviours, both development and implementation. Policy efforts can be directed at the municipal level (eg, by-laws) and/or the institutional level (eg, school or worksite policy).
  - Steps taken to plan for and engage appropriate partners (eg, municipal politicians, decision makers) to develop a policy.
  - Appropriate partners are engaged (ie, strong alliances established to address policy development and implementation). A policy change plan is in place.
  - All of 3 above and action taken to operationalize strategy to promote policy change.
**Level:**
**Rationale:**
**Suggestions for Enhancement:**
  An experienced reviewer can complete a program assessment in 90 minutes. Someone new to the assessment process may need 3 to 4 hours. How well the program information survey is completed also factors into the time required to complete an assessment.

**Figure 3.** The program assessment worksheet helps reviewers rank the program against the 19 review criteria and record the rationale for the ranking and suggestions for improvement. This excerpt is for criteria 3, Theory and Literature Evidence.

### Step 4: Consensus meeting

**Figure 4:**
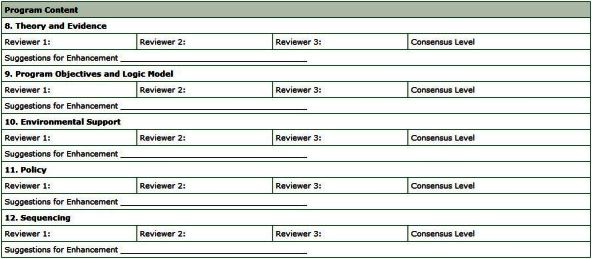
The consensus summary worksheet summarizes the rankings of individual reviewers and their suggestions for enhancement, according to the 19 review criteria, as exemplified here for criteria 3 and 4.

The consensus meeting provides an opportunity for program reviewers and stakeholders to establish a final rating for each criterion and to generate, through a dialogue of multiple perspectives, final suggestions to improve the program. Before the meeting, input from each program reviewer is collated via the consensus summary worksheet and distributed to all participants. The final consensus ratings and suggestions for program enhancement are also documented on this worksheet (Figure 4).

A successful consensus meeting requires that all participants feel they have equal opportunity to contribute to the discussion. We have found that assigning a consensus meeting facilitator is useful in this regard. This role is also charged with keeping discussions to within the established timeline, usually 90 minutes, and ensuring that all 19 criteria are addressed.

The consensus meeting is perhaps the most critical aspect of a program assessment. Our experience has shown that participating in these discussions accounts for much of the learning and capacity building that occurs throughout the program assessment process.

### Step 5: Select and implement suggestions

**Figure 5:**
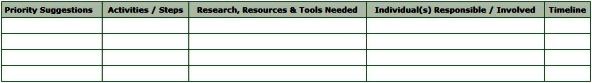
The program enhancement work plan is used to summarize the actions, resource needs, accountability and timelines for each priority for program enhancement.

Program stakeholders decide which, if any, of the suggestions for program enhancement are implemented. Decisions are based on factors such as resource availability, political acceptability, and organizational support, all of which are context dependent. Thus, the final step in a program assessment involves program stakeholders meeting to select priorities and to develop feasible plans for program enhancement. Final decisions are documented on the program enhancement work plan (Figure 5).

The suggested timeline to complete all 5 steps in a program assessment is 7 weeks. The actual time required of participants depends on their role or roles, other work responsibilities, and their experience in conducting assessments. The first assessment is the most time consuming, as participants spend time understanding the process and the criteria.

## Using the TEIP Program Assessment Tool

Since 2005, the TEIP Program Assessment Tool has been applied to municipal public health programs in Ontario and to health promotion initiatives where public health practitioners partner with a community-based coalition. Examples of initiatives applying the tool include smoking prevention and cessation, elementary school health promotion, active transportation, blood pressure education, pedometer lending libraries, grade 9 healthy heart screening, stroke prevention, and various workplace wellness initiatives. The tool is not as good a fit for the more clinically oriented and hospital-based programs (ie, stroke prevention clinic) where health promotion principles are not well embedded into the culture of practice. However, use of the tool in these contexts can lead to increased understanding of health promotion concepts and approaches among clinic staff.

We conducted a TEIP Program Assessment of an adult smoking cessation program delivered at a small health unit serving a largely rural population. The smoking cessation program consisted of 4 weekly sessions led by a facilitator and an optional monthly support group. The program was created on the basis of a promising practice developed for low-income women at another health unit 10 years earlier. Program materials had not been revised in many years and looked dated, although the information and the concepts themselves were sound. Enrollment was small, 6 to 8 people per session, despite the region having among the highest rates of smoking in the province. Some sessions were cancelled because of insufficient enrollment. Evaluation of the program consisted mainly of satisfaction-type questions, the results of which were never collated or used to influence program decisions. Program staff avoided asking outcome-related questions out of fear the program would be unfairly deemed unsuccessful since it was generally known that most individuals require multiple quit attempts before achieving success.

During the consensus meeting, suggestions for enhancement focused on several areas: 1) update the look of program materials to be more modern and appealing, 2) streamline program registration to collect baseline and follow-up data to measure interim outcome indicators toward quitting (ie, smoking fewer cigarettes per day, delaying the first cigarette of the day, smoking in fewer locations), and 3) increase marketing efforts by drawing on partners in the hospitals and stroke clinics to advertise their program.

The program team took the suggestions for enhancement seriously. They invested in the services of a marketing consultant. The program was rebranded as the iQuit Program, and an eye-catching logo and new program materials were developed. The health unit’s epidemiologist assisted in developing a preintervention, postintervention, and 6-month follow-up evaluation to assess interim outcome indicators for smoking cessation, drawing on indicators found in peer-reviewed literature. A marketing plan was developed to better promote their program across the region.

The program assessment breathed new life into a tired and underappreciated program. The refreshed program was presented at provincial smoking cessation conferences and was nominated for a local health promotion innovation award. Three years later, the program coordinator continued to review the original suggestions from the consensus committee for additional program enhancement ideas.

## Benefits and Challenges

Feedback on use of the Program Assessment Tool was obtained from a formative evaluation conducted during 2005 and 2006, and from approximately 300 participants who attended TEIP Master Trainer workshops from 2008 through 2011.

Benefits of using the Program Assessment Tool have been reported at the individual, program, and organizational levels. At the individual level the process is seen as particularly helpful in increasing understanding and skills in the application of best practice concepts. Practitioners appreciate the focus on program enhancement in the assessment process and the supportive context of the review. They also report increased knowledge and interest about program evaluation. At the program level, the structured review process is reported as useful in strengthening planning processes for any chronic disease prevention program. Many participants report that the program information survey provides an opportunity to improve program documentation and that the overall assessment process provides suggestions to strengthen program evaluation activities. At the organizational level, the tool is reported to strengthen collaboration between other groups engaged in chronic disease prevention programming through involving partners and collaborators in the assessment process and in consensus meetings.

The most consistent challenge cited by participants is the substantial time and resource constraints under which they work. In response, alternative applications of the Program Assessment Tool have been explored, including assessing 1 aspect of a program such as evaluation needs or program content, using the review criteria as a checklist in planning new programs or in conducting a mini-program review, using the assessment process to initiate relationships with new partners, and using the criteria to set planning goals for future program achievement. Another common challenge is the reported lack of adequate documentation of the local programs needed to complete the review process.

## Discussion

Creating relevant opportunities for knowledge exchange and deliberative dialogue is essential given the array of evidence and information available (23,24). The Program Assessment Tool fosters a unique opportunity for consultation among stakeholders for consideration of various types of evidence. It also offers an opportunity to bring outside expertise into the program planning process, providing constructive feedback for the enhancement of local public health programs. Users reported the discussion to be a forum in which multiple sources of evidence and viewpoints are valued and debated.

Just as important as creating space for knowledge exchange is the content of the dialogue and discussion. The TEIP Program Assessment Tool provides a process that emphasizes continual program improvement and using research as well as experiential knowledge to support decision-making. The tool and related supports meet public health practitioners where they are, helping to build on existing program design and implementation strengths. The process also acknowledges the complexity of community-based programs, organizational and management structures, and available resources. The combined opportunity for meaningful knowledge exchange and the emphasis on program enhancement empowers public health practitioners to be more confident in making program-related decisions and reduces apprehension about review of programs for the purposes of ongoing improvement. As a user observed, “[It is] important to put the criteria out there and people will rise to the bar.”

Provision of capacity building and leadership are particularly important in maintaining momentum and enthusiasm for the assessment process in the implementation of effective and robust chronic disease prevention programs (25,23). Thus, system-level pressures to encourage use of continuous improvement tools in public health are critical. For example, the National Voluntary Accreditation for Public Health Departments program (26) launched in September 2011 aims to “improve the health of the public by advancing the quality and performance of all public health departments.” Such bodies can recommend and give credit to health departments that embed use of quality improvement processes such as the TEIP Program Assessment Tool. In Ontario, public health departments are using aspects of the tool to meet public health standards for program assessment and knowledge exchange (27). More broadly, the Public Health Agency of Canada has acknowledged benefits of the tool by promoting its dissemination to other Canadian provinces and translating the tool into French.

The TEIP Program Assessment Tool contributes to the use of evidence-informed practice through reinforcing and supporting the use of best practices by focusing on strengthening better processes and ongoing program improvement. The impact of the TEIP Program Assessment Tool will only be realized with adequate organizational support and leadership at multiple levels. Public health leaders at multiple levels need to champion and support the dissemination and use of the tool, including ongoing improvements over time. Future work should assess the extent to which the use of the tool affects longer-term outcomes at the practitioner, program, and organizational levels. Accreditation bodies can acknowledge and give credit to organizations employing continuous improvement processes such as the TEIP Program Assessment Tool. The result will be improved public health and chronic disease prevention activities.
